# Innovation In Gerontology Education: An Adventure of Grand Ideas and Good Intentions

**DOI:** 10.1093/geroni/igaa057.1773

**Published:** 2020-12-16

**Authors:** Dana Bradley

**Affiliations:** University of Maryland Baltimore County, Baltimore, Maryland, United States

## Abstract

Gerontology educators continually innovated over multiple decades to create a field characterized by its diversity of programs with a variety of structures to meet a wide range of educational experiences. This paper explores the rich history of how innovations emerged and shares insights on how these grand ideas became broadly accepted practice. Since early 1970’s 6 distinct innovations emerge with sticking power, including convergence upon a core intellectual foundation; support of interdisciplinary education; offering internships or practicums; integration of gerontological training with opportunities provided by community/organizational partners, research affiliates, and alumni to foster applied learning experiences in research, policy, and practice; and, development of leadership across and among generations through the Emerging Scholar & Professional Organization (ESPO).

